# An Open Label Pilot Study of a Brief Psychosocial Intervention for Disaster and Trauma Survivors

**DOI:** 10.3389/fpsyt.2020.00483

**Published:** 2020-06-26

**Authors:** Meaghan Louise O'Donnell, Winnie Lau, Julia Fredrickson, Kari Gibson, Richard Allan Bryant, Jonathan Bisson, Susie Burke, Walter Busuttil, Andrew Coghlan, Mark Creamer, Debbie Gray, Neil Greenberg, Brett McDermott, Alexander C. McFarlane, Candice M. Monson, Andrea Phelps, Josef I. Ruzek, Paula P. Schnurr, Janette Ugsang, Patricia Watson, Shona Whitton, Richard Williams, Sean Cowlishaw, David Forbes

**Affiliations:** ^1^ Phoenix Australia Centre for Posttraumatic Mental Health, The University of Melbourne, Carlton, VIC, Australia; ^2^ Department of Psychiatry, University of Melbourne, Carlton, VIC, Australia; ^3^ School of Psychology, University of New South Wales, UNSW Sydney, Kensington, NSW, Australia; ^4^ Division of Psychological Medicine and Clinical Neuroscience, Cardiff University, Cardiff, United Kingdom; ^5^ Australian Psychological Society, Melbourne, VIC, Australia; ^6^ Department of Psychiatry, Combat Stress, UK, Leatherhead, United Kingdom; ^7^ Australian Red Cross, North Melbourne, VIC, Australia; ^8^ Mental Health Promotion and Illness Prevention, Addiction Mental Health - Alberta Health Services, Calgary, AB, Canada; ^9^ Academic Department of Military Mental Health, King's College London, London, United Kingdom; ^10^ College of Medicine and Dentistry, James Cook University, Douglas, QLD, Australia; ^11^ Centre for Traumatic Stress Studies, The University of Adelaide, Adelaide, SA, Australia; ^12^ Department of Psychology, Ryerson University, Toronto, ON, Canada; ^13^ National Center for PTSD, Dissemination and Training Division, US Department of Veterans Affairs, Palto Alto, CA, United States; ^14^ Department of Psychiatry and Behavioral Sciences, Stanford University, CA, United States; ^15^ National Center for PTSD, Executive Division, US Department of Veterans Affairs, White River Junction, VT, United States; ^16^ Department of Psychiatry, Geisel School of Medicine, Dartmouth, Hanover, NH, United States; ^17^ Asian Disaster Preparedness Center, Bangkok, Thailand; ^18^ Welsh Institute for Health and Social Care, University of South Wales, Wales, United Kingdom; ^19^ Bristol Medical School, University of Bristol, Bristol, United Kingdom

**Keywords:** trauma, adjustment disorder, posttraumatic stress, disaster, psychosocial intervention, brief intervention, sub-clinical, sub-syndromal

## Abstract

**Background:**

In the aftermath of disaster, a large proportion of people will develop psychosocial difficulties that impair recovery, but for which presentations do not meet threshold criteria for disorder. Although these adjustment problems can cause high distress and impairment, and often have a trajectory towards mental health disorder, few evidence-based interventions are available to facilitate recovery.

**Objective:**

This paper describes the development and pilot testing of an internationally developed, brief, and scalable psychosocial intervention that targets distress and poor adjustment following disaster and trauma.

**Method:**

The Skills fOr Life Adjustment and Resilience (SOLAR) program was developed by an international collaboration of trauma and disaster mental health experts through an iterative expert consensus process. The resulting five session, skills-based intervention, deliverable by community-based or frontline health or disaster workers with little or no formal mental health training (known as coaches), was piloted with 15 Australian bushfire survivors using a pre-post with follow up, mixed-methods design study.

**Results:**

Findings from this pilot demonstrated that the SOLAR program was safe and feasible for non-mental health frontline workers (coaches) to deliver locally after two days of training. Participants' attendance rates and feedback about the program indicated that the program was acceptable. Pre-post quantitative analysis demonstrated reductions in psychological distress, posttraumatic stress symptoms, and impairment.

**Conclusions:**

This study provides preliminary evidence that the delivery of the SOLAR program after disaster by trained, frontline workers with little or no mental health experience is feasible, acceptable, safe, and beneficial in reducing psychological symptoms and impairment among disaster survivors. Randomized controlled trials of the SOLAR program are required to advance evidence of its efficacy.

## Introduction

It is well established that disasters of both natural (e.g., floods, bushfires, earthquakes) and human (e.g., mass violence, terrorism) origin can adversely impact mental health among those directly or indirectly exposed (1). A range of psychiatric disorders can develop in the aftermath of disaster exposure, including alcohol use disorder, posttraumatic stress disorder (PTSD), obsessive–compulsive disorder, generalized anxiety disorder, and major depressive disorder (2). Alongside losses, hardships, and other psychosocial stressors endured, these conditions not only cause great personal suffering and distress, but also interfere with family, social, and occupational functioning (3). The costs of the mental health consequences of disaster to the community in both human and financial terms is therefore enormous, and is currently recognized by global agencies as one of the most urgent public health issues (4).

In the short to longer-term aftermath of disaster, there is consensus for supporting a strategic, stepped model of care comprising universal, indicated, and standard treatment components (5). Approaches informed by Psychological First Aid (PFA) are often recommended as early universal intervention strategies, and are designed to foster cohesive, informational, practical, and mutual support (6, 7); although it is recognized that little research has confirmed that PFA approaches actually achieve these goals (8, 9). At the other end of the spectrum, substantial research exists to guide evidence-based psychological and pharmacological interventions for disaster survivors who present with diagnosable psychiatric conditions, such as posttraumatic stress disorder (10). However, significant knowledge and practice gaps exist as to how best to assist the substantial number of people who develop disabling and distressing adjustment problems, or sub-clinical psychiatric conditions after disaster, that do not reach clinical thresholds for psychiatric diagnosis. Targeting interventions to this population through indicated interventions is essential for three key reasons: (i) there is evidence that most mental health difficulties following disaster are of a mild-to-moderate (i.e., subclinical) severity (11), (ii) psychological dysfunction at this level can cause significant distress, functional impairment, and economic loss (12), and (iii) these adjustment problems pose a risk for escalation into serious psychiatric disorders if not effectively addressed (13).

Scalability is an important issue when devising post-disaster psychosocial interventions. Such interventions need to be deliverable to potentially large numbers of people across diverse settings, and there is typically insufficient capacity to achieve this using existing mental health resources. One way to improve scalability is to design brief interventions, which are preferable for implementation purposes, as brevity minimizes the costs of delivery and reduces the burden on participants (14). “Task–shifting” is a feature of many recent, scalable, psychosocial initiatives, which moves delivery of interventions from mental health specialists to less qualified or trained personnel. Recent meta-analyses indicate that the use of non-specialists can lead to significant improvements in mental health (15).

There is growing evidence of the efficacy of brief, low-intensity interventions and their capacity to meet existing gaps in health care for persons with subclinical disorders. The Improving Access to Psychological Therapies (IAPT) stepped model of care, for example, introduced in the United Kingdom in 2008, incorporates low-intensity interventions delivered by psychological wellbeing practitioners (PWPs) for persons with mild-to-moderate depression and anxiety. PWPs, who do not typically belong to a mental health profession such as clinical psychology, social work, or mental health nursing, are trained to deliver the core IAPT low-intensity interventions and to act in the role of coaches. This approach has greatly expanded the proportion of UK residents benefitting from NICE recommended psychological interventions, and has substantially reduced waiting times for services (16, 17). Taking a similar approach to low and middle-income countries, the World Health Organization (WHO) developed the brief scalable intervention, Problem Management Plus (PM+) (18, 19). PM+ is a five-session intervention that targets common mental health disorders such as depression or anxiety. PM+ has been subjected to randomized controlled trials in Pakistan (20) and Kenya (21), and in both cases has been shown to significantly improve mental health outcomes.

Despite these promising outcomes, there remains uncertainty regarding the capacity for scalable, low-intensity interventions to meet the needs of trauma survivors specifically. Few attempts have been made to develop interventions targeted specifically to disaster and/or trauma survivors, with one exception being the Skills for Psychological Recovery (SPR) program (22), a flexibly-delivered six-module intervention program delivered by generalist health providers or paraprofessionals. However, SPR does not include any emotional processing component, which has been found to be a frontline strategy for addressing posttraumatic emotional reactions (23). Notably, emotional processing is also not featured in the IAPT low-intensity interventions or PM+. Further, SPR has been critiqued as too complex for non-mental health professionals to deliver in practice (23). SPR is also limited by the absence of any published trials examining its efficacy since its development over a decade ago for disaster survivors following Hurricane Katrina in the USA.

Recognizing the gap in scalable, simple interventions that cater for the psychosocial needs of disaster and trauma survivors experiencing adjustment and subclinical mental health problems, an international group of experts were assembled to develop a new intervention: the Skills fOr Life Adjustment and Resilience (SOLAR) program. This paper describes the development, structure, and psychosocial treatment components of SOLAR, along with the findings of a pilot study assessing the feasibility of training non-mental health professionals and paraprofessionals to deliver SOLAR, and the safety and acceptability of the program among bushfire survivors in Australia.

## Method

### Program Description

#### SOLAR Development

The process for developing SOLAR was informed by the UK Medical Research Council (MRC) guidelines for developing a complex intervention (24). It entailed an iterative expert consensus process involving: (i) the formation of an international expert group; (ii) a scoping process to identify relevant literature concerning mechanisms of trauma recovery and evidence-based interventions; (iii) a subsequent, iterative ranking process to identify and weight treatment components suitable for inclusion in the SOLAR program; (iv) a roundtable meeting of experts to enable final consensus on program components.

##### Formation of an International Expert Group

A SOLAR Development Group was established comprising 21 international experts in trauma or disaster mental health, and/or disaster response, from the USA, UK, Canada, Australia, and Asia. Experts were selected based on their profile expertise in disaster and mental health, evidenced by publication records, demonstrated impacts in their fields, and/or senior level experience and expertise in emergency or mental health disaster response. The SOLAR Development Group also included expert representation from frontline disaster response agencies, including the Australian Red Cross and the Asia Disaster Preparedness Center, to ensure the relevance and feasibility of the intervention.

##### Literature Scoping to Identify Mechanisms of Recovery

A subgroup of the SOLAR Development Group (the Scientific Working Party) reviewed and identified empirical literature to determine mechanisms central to trauma recovery and components of evidence-based therapeutic approaches aligned with each recovery mechanism. In doing so, consideration was given to therapeutic approaches that focused on skills-development. The following mechanisms were identified and formed the basis for consensus building among the SOLAR development group: (1) managing arousal and distress/affect regulation [e.g., (25–30)]; (2) emotional processing and managing avoidance [e.g., (29, 31–38)]; (3) social support [e.g., (3, 39–45)]; (4) problem solving [e.g., (46, 47)]; (5) cognitive restructuring/control [e.g., (36, 48–52)]; (6) psychoeducation [e.g., (53–56)]; (7) activity scheduling and behavioural activation [e.g., (7, 46, 48, 57, 58)]; (8) healthy living and self-care [e.g., (7, 59)]; and (9) mindfulness [e.g., (26, 28)].

##### Iterative Ranking Process

A summary of findings from the literature scoping process was circulated to the SOLAR Development Group, who provided comment and ranked components of the identified skill-based therapeutic approaches according to their suitability for SOLAR. Experts were also able to suggest additional therapeutic approaches for the Group's consideration. When ranking, the Development Group sought a balance between (i) efficacy and scalability, recognizing that this intervention needed to be effective while also being deliverable in a short time-frame by generalist and lay providers, and ii) specificity and generalizability, with the aim being to devise a program with universal application across disaster and potentially other large-scale traumatic events. The ranking process was repeated twice. After each ranking, the Scientific Working Party revised the intervention proposal and provided commentary on the level of consensus and rationale for the inclusion or exclusion of each component, according to expert feedback.

##### International Roundtable Meeting

**Figure 1:**
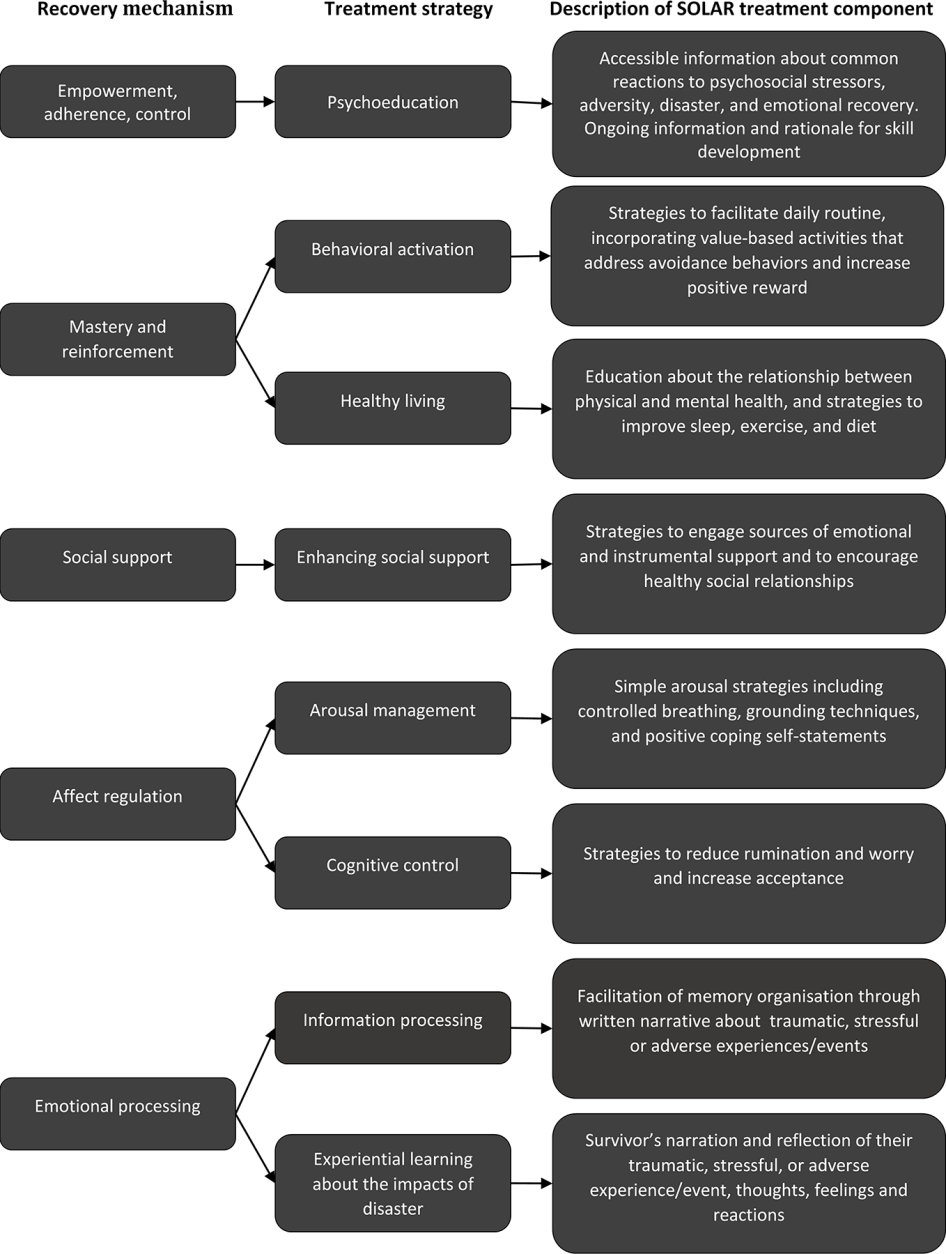
The SOLAR program: Mechanisms of post-trauma and mental health recovery, relevant treatment strategies, and summary description of SOLAR treatment components.

The consensus process culminated in a roundtable meeting held in Sydney, Australia in 2015, during which the SOLAR Development Group achieved consensus on the therapeutic components to be included in SOLAR, a preferred delivery format, and an approach for its evaluation. The decision-making process occurred with reference to the findings of the literature scoping and ranking process described above. The components included in the final program, together with the recovery mechanism they target, are shown in **Figure 1**. 

Following the roundtable meeting the SOLAR content was developed and circulated to all those in the SOLAR Development Group for their input.

#### SOLAR Structure and Treatment Components

A workshop was conducted with end-users to present the beta version of SOLAR for input and discussion. The key focus of this consultation forum was to get feedback as to whether the information was in a format that could be easily understood and delivered by non-mental health experts. The resulting intervention program, SOLAR, constitutes a 5-session psychosocial intervention that targets subclinical psychiatric symptoms and adjustment difficulties in the medium-to-long term following disaster or trauma. It represents an intervention that lies between universal interventions and specialized interventions for individuals with psychiatric diagnoses, to enable a strategic, scalable, stepped model of post-trauma mental healthcare. The program comprises six modules, outlined in **Table 1**.

**Table 1:** SOLAR program modules.

Module	Description	Component skills and activities
Healthy living	Highlights the interrelationship between physical and mental health and the risks that disaster and associated disruptions to lifestyle pose for health. Explores ways to improve diet, increase physical activity, and improve sleep quality.	Strategies to promote quality sleep Building a routine that incorporates physical activity Habits to create and maintain a healthy diet
Managing strong emotions	Introduces strategies for managing anxiety and everyday physiological symptoms of stress.	Controlled breathing to reduce arousal Using a Subjective Units of Distress (SUDS) rating scale to self-monitor arousal ‘Here and now’ grounding exercises to connect to the present moment
Getting back into life	Describes the importance of routines and engagement in purposeful, value-based activities, as well as the likelihood of disruption and disengagement following disaster or trauma. Encourages re-engagement in a range of valued activities and introduces problem solving to overcome barriers that limit engagement.	Identifying personal values and goals Creating an activity plan Problem-solving barriers to implementing valued activities
Coming to terms with the disaster	Focuses on making sense of the disaster event and organizing disjointed traumatic memories into a coherent narrative.	Using narration to consolidate a coherent traumatic memory and reduce traumatic stress symptoms
Managing worry and rumination	Investigates the impacts of worry and rumination on mood and behaviour, and introduces strategies to manage these problematic thinking patterns.	Psychoeducation about negative, repetitive thinking Using distraction to interrupt cycles of worry and rumination Introducing structured ‘worry/rumination' time to minimize their adverse impacts on daily life Using problem solving and logic to counter worry and rumination
Maintaining healthy relationships	Identifies common impacts of trauma on relationships and explores methods for managing stress and conflict in interpersonal relationships.	Finding opportunities for shared participation in valued activities Distinguishing assertive, aggressive, and passive forms of communication Collaborative problem-solving to resolve conflict

The SOLAR program is designed for delivery by volunteers, professionals, or paraprofessionals working in health or disaster response in communities affected by disaster. These providers, termed ‘coaches’ to program participation or practice, complete a 2-day training program before commencing the program with identified disaster/trauma survivors. Coaches are not expected to have specialist expertise in mental health, which increases the program's potential reach and helps to prevent overwhelming an already burdened mental healthcare system in the aftermath of disaster.

The role of coaches is to teach recovery skills, maintain motivation, encourage practice, reinforce effort, and problem-solve barriers to program participation or practice with participants. Following training, coaches are provided with a manual, and encouraged to work sequentially through the manualized program with a participant. Supervision is offered weekly, recognizing the critical role of on-going supervision for developing the skill base of non-mental health specialists. Supervision also assists with the identification of participants requiring more intensive or specialized treatment, consistent with a stepped-care approach (21). The supervision schedule may be varied once the coach completes the program with at least two participants and is regarded as competent by the supervisor.

Each SOLAR session runs for 50 min, with the exception of the first, 80-minute session. Sessions are delivered to participants face-to-face on a weekly basis, and can be provided at any easily accessible location in the community. Skills that the SOLAR Development Group prioritized for early gain are frontloaded, to avoid participants missing this information in the event of subsequent non-attendance or attrition, which is common in transient disaster populations. Maintenance of skill development beyond each session is promoted through practice tasks, which the participant completes between sessions. Each session also includes revision of the skills taught previously, and the final session constitutes a review of learning overall and the development of a plan for continued recovery into the future. Participants are given a workbook summarizing the content delivered in each session, which provides a clear rationale for undertaking practice tasks, and includes worksheets to help them complete tasks and monitor progress.

### Study Design

To assess the safety, feasibility, and acceptability of SOLAR, a single group study was conducted. Assessments were conducted at pre-intervention, post-intervention, and at 3-month follow-up. All eligibility screening and assessments were conducted by a trained research assistant *via* telephone. The study was conducted in 2016 with survivors of the January 2015 Sampson Flat bushfires and the November 2015 Pinery bushfires in South Australia. The trial took place in partnership with Country South Australia Primary Health Network (CSAPHN), the Northern Health Network (NHN), and the Australian Red Cross. Ethics approval was provided by the Health Sciences Human Ethics Sub-Committee of the University Of Melbourne. All participants underwent an informed consenting procedure and signed an informed consent form prior to participating.

#### Coach Recruitment, Training, and Supervision

Seven frontline workers were nominated by local partnering organizations and trained as coaches. They consisted of a community nurse, an intern social worker, two case workers, and three Australian Red Cross volunteers. Coaches completed a 2-day training workshop delivered by registered psychologists with trauma expertise. Coaches were provided with the SOLAR coach manual and participant workbook, as well as access to a “web-hub” developed to facilitate communication between coaches and supervisors, and to enable access to shared resources. Coaches' knowledge and understanding of the material, and their confidence in delivering SOLAR, was measured before and after the training workshops using a purposively designed, 14-item test. Readiness to act as a SOLAR coach was defined as a minimum of 80% correct in the knowledge items, and a minimum of 70% in confidence. Coaches attended weekly group supervision *via* teleconferencing for a minimum of two participants and until deemed competent in delivering SOLAR.

#### Participant Recruitment

**Figure 2:**
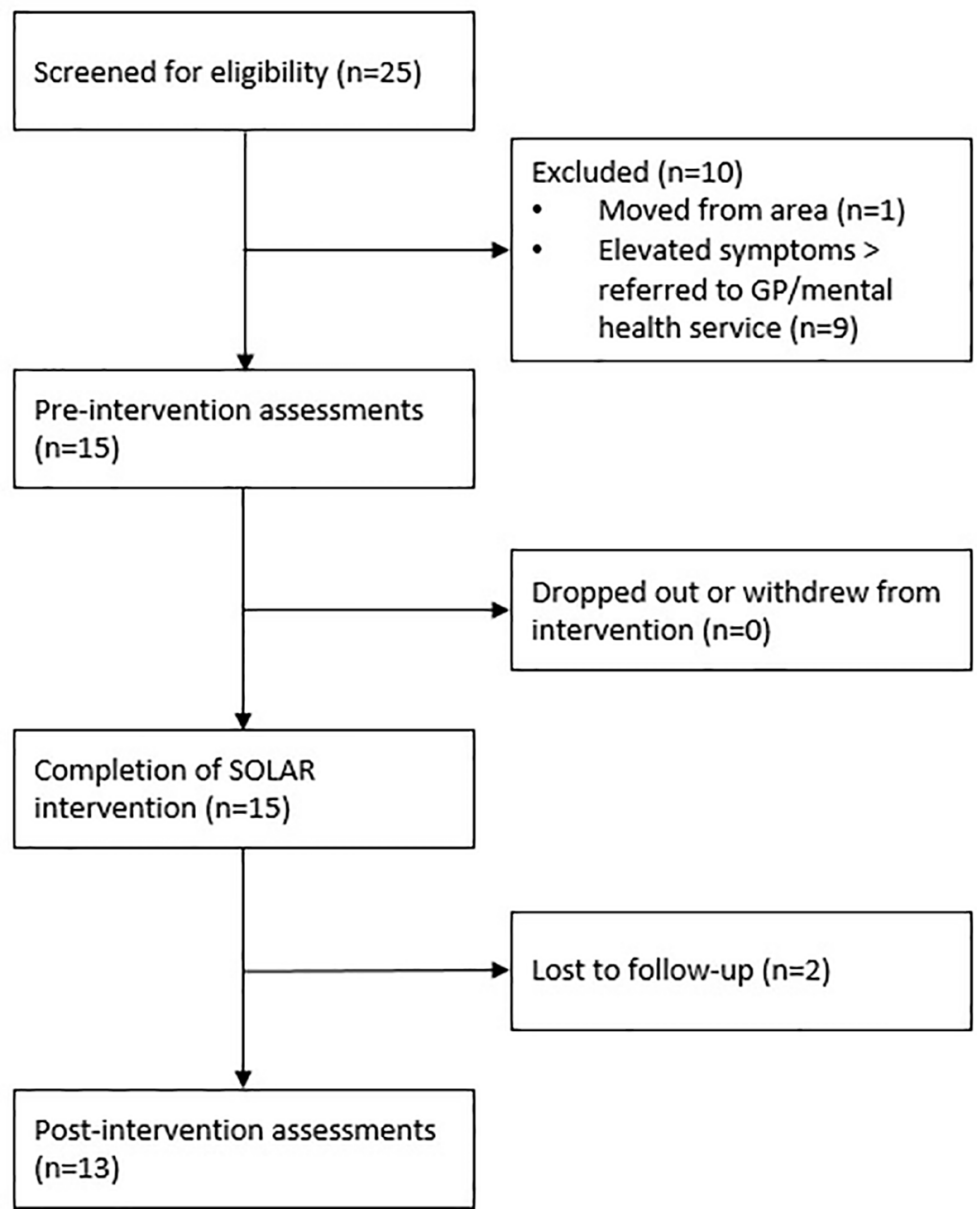
The flow of participants recruited to the SOLAR pilot study.

Fifteen participants were recruited over a six-month period *via* referral from local partnering organizations, or *via* self-referral in response to trial promotion materials. Trial promotional material was disseminated through local Red Cross bushfire-related activities, and through local general practitioner clinics. **Figure 2** provides details of participant recruitment, screening, assessment, and program participation. Eligibility criteria were: (i) ≥18 years of age, (ii) directly experienced or impacted by the 2015 Sampson Flat or Pinery bushfires, according to self-report, (iii) subclinical anxiety, posttraumatic stress, or depression symptoms, as determined by cut-off scores on assessment measures, (iv) distress and impairment in social, occupational, or daily functioning, (v) no previous or current diagnosis of psychiatric disorder, as determined by structured clinical interview, and (vi) availability for the program and not participating in other mental health treatments. Participants who were excluded because of severe distress or psychiatric symptoms were referred to an appropriate mental health service. Eligible participants were allocated to a coach based on location and availability. Sample demographic information is presented in **Table 2**.

**Table 2:** Pilot sample characteristics (n = 15).

Sample Characteristics	*N*	%	*M*	*SD*
Female (yes)	8	53.3		
Age (range: 39–74)	–	–	58.68	11.53
Employed (yes)	5	41.7	–	–
Property was damaged (yes)	9	75	–	–
Undergoing insurance claim (yes)	9	75	–	–
Main Presenting Problem—PSYCHLOPS				
General Health Concerns	5	33	–	–
Relationship Concerns	5	33	–	–
Daily Stressors	5	33	–	–
Extent affected by on-going stressors related to fires^a^			6.75	2.18

a Range: 1 = Not at all, 10 = To a great extent.

### Assessment Measures

*The Kessler Psychological Distress Scale (K10)* (60) was used as a screening measure of global psychological distress to determine inclusion into the pilot. The K10 comprises 10 items that rate symptoms along the anxiety–depression spectrum, with a five point Likert response option for each item, where 1 = symptom experienced not at all, and 5 = symptom experienced all the time. A score on the K10 <30, with at least two symptoms endorsed, was used to determine inclusion. The K10 was also used as a pre and post-intervention measure and follow-up measure of global psychological distress. The K10 has high levels of discriminant and criterion validity (61), as well as convergent validity with other measures of psychological distress (62). It also consistently shows very good levels of internal reliability as measured by Cronbach's alpha (63).

*The PTSD Checklist for DSM-5 (PCL-5)* (64) was also used to screen and determine eligibility into the pilot. The PCL-5 comprises 20 items with 5-point Likert response options, where 0 = symptom experienced not at all, and 4 = symptom experienced extremely. A score <33, with at least two symptoms endorsed was used to determine inclusion. The PCL-5 was also used as a pre and post-intervention and follow-up measure of posttraumatic stress symptoms. The PCL-5 has demonstrated high levels of internal consistency, as well as convergent, discriminant, and structural validity (65).

*Impairment* was measured using a single-item question developed by the SOLAR project team to provide an assessment of functional impairment in major life areas, including work or study, social activities, relationships, tasks of daily care, or other area (i.e., How much did the things you describe cause you distress and affect your ability to function in your work, your relationships with other people, and in other important areas of your life?). The item was rated using a ten-point Likert response scale, where 0 = none and 10 = extreme. This item was also used as a pre and post-intervention and follow-up measure of impairment. Participants were only included in the pilot if they met screening eligibility according to K10 and PCL-5 thresholds, in addition to endorsement of the impairment item.

*The Mini International Neuropsychiatric Interview Plus 7 (MINI Plus 7)* (66) was used as a diagnostic screening tool to exclude participants with a psychiatric diagnosis from the pilot. The MINI Plus 7 is a clinical assessment tool that assesses the presence of psychiatric disorders using DSM-5 symptom criteria, rated using a dichotomous Yes/No response option. The following modules were employed: (1) PTSD, (2) major depression, (3) panic disorder, (4) agoraphobia, (5) social anxiety disorder, (6) general anxiety disorder, (7) alcohol use disorder, and (8) substance use disorder. The MINI has strong psychometric properties, with good inter-rater and test–retest reliability (66). Specificity is above .70 for all diagnoses used in this study, and sensitivity is above .70 for all modules used except for agoraphobia (66).

*The Psychological Outcome Profiles instrument (PSYCHLOPS)* (67) was used to measure participant-generated outcomes. It comprises four items that assess outcomes generated by the client on main problems they are presently experiencing, functioning, and wellbeing, as well as how much clients are affected by the problems they are experiencing. The PSYCHLOPS was used to assess outcomes perceived as important by participants regarding their difficulties, and as a measure of change pre and post-intervention and at follow-up. The PSYCHLOPS is sensitive to clinical change after therapy and has satisfactory levels of internal reliability, as well as convergent, concurrent, and construct validity (68).

### Feasibility

Assessment of feasibility concerned the achievability of training frontline disaster workers without prior formal mental health training to deliver the SOLAR program following a 2-day workshop. This was determined by changes in pre-post training in coaches' knowledge of the intervention, which was assessed using a 14-item multiple choice questionnaire (four response options for each item), and by changes in coaches' confidence in providing the intervention, which was assessed using an 8-item questionnaire rated on a 5-point scale, where 0 = not at all confident and 4 = very confident. Outcome criteria for feasibility was defined as a minimum of 80% correct in the knowledge items, and a minimum of 70% in confidence.

### Acceptability

The acceptability of SOLAR was assessed by the number of sessions completed by participants and participants' responses to two, open-ended questions included in a self-report questionnaire at post-intervention assessment, (i) ‘How useful did you find the SOLAR program overall?', (ii) ‘Would you recommend this program to others struggling after a disaster?'.

### Safety

Safety was determined by monitoring for adverse events and assessing symptom measure trajectories from pre to post-intervention and at three-month follow-up. The K-10 (61) was used to assess psychological distress, the Posttraumatic Stress Disorder Checklist for DSM-5 [PCL-5, (65)] was used to assess posttraumatic stress symptoms, and the Psychological Outcomes Profiles [Psychlops, (69)] was used to assess functioning.

### Data Analyses

A Wilcoxon signed-rank test was used to examine change in coaches' knowledge of the intervention and confidence in delivering it pre to post-training. Repeated measures effect size estimates (dRM) with 95% Confidence Intervals (CIs) were produced to quantify the magnitude of participant within-group change in each outcome, from (a) pre to post-intervention, and (b) post-intervention to follow-up. These were based on formulas for the single-group pretest–posttest design, which standardize the sample mean change by variability in change scores (70). Data preparation was conducted in SPSS Version 23, while the dRM estimates were produced in Program R (version 3.1.3) using the Package ‘effsize’ (4). Individual trajectories were also produced using the Package ‘ggplot2’ (71), and were displayed graphically to contextualize the group estimates and explore the variability in individual scores over time.

## Results

### Feasibility

Analyses revealed a significant improvement in coaches' knowledge of the program from pre-training (Mdn_pre_ = 11) to post-training (Mdn_post_ = 14; Z = −3·20, p = 0·001), and a significant improvement in coaches' confidence to deliver the program (Mdn_pre_ = 24, Mdn_post_ = 35, Z = −3·18, *p* = 0·001). All coaches met the readiness criteria.

### Acceptability

All participants determined to be eligible for the program agreed to participate. Of these 15 participants, all completed the total number of sessions, suggesting a high level of program acceptability. Six participants completed a self-report questionnaire concerning their satisfaction with the program. Of these, all reported that they had found the program useful, and that they would recommend the program to other disaster survivors.

### Safety and Effectiveness

No adverse events were reported and symptoms did not deteriorate in the three months after the SOLAR program. Participants' scores on the K-10, PCL-5, and PSYCHLOPS are shown in **Table 3**, which includes descriptive statistics (Means, SDs) at pre-intervention, post-intervention, and follow-up, as well repeated measures effect size estimates (d_RM_). Missing data was managed through pairwise deletion.

**Table 3:** Symptom score statistics and repeated measures effect size estimates (d_RM_) on K10, PCL-5 and PSYCHLOPS from pre-test to post-test and from post-test to follow up.

Outcomes	Pretest			Posttest			Follow up			*d_RM_*					
	*n*	*M*	*SD*	*n*	*M*	*SD*	*n*	*M*	*SD*	Pretest – Posttest			Posttest - Follow up		
										Estimate	95% CI		Estimate	95% CI	
											LB	UB		LB	UB
**K-10**	15	18.40	5.01	13	13.08	2.36	15	13.73	2.81	−1.18	−2.06	−0.31	0.16	−0.65	0.97
**PCL-5**	15	17.87	8.29	14	5.07	5.65	14	6.93	6.51	−1.82	−2.74	−0.90	0.32	−0.50	1.13
**PSYCHLOPS**	14	11.79	4.39	12	5.25	2.30	12	5.67	2.84	−1.79	−2.79	−0.78	0.12	−0.73	0.97
**Impairment**	14	4.64	1.95	14	1.07	1.54	15	2.2	2.27	−1.56	−2.48	−0.63	0.49	−0.30	1.28

PCL, Posttraumatic Stress Disorder Checklist, K-10, Kessler 10, PSYCHLOPS, Psychological Outcomes Profiles, d_RM_, effect size estimates, CI, confidence intervals, LB, lower boundary, UP, upper boundary.

Descriptive statistics indicated reductions in mean scores for all outcomes from pre to post-intervention, along with a slight increase from post-intervention to follow-up. These interpretations were supported by the d_RM_ estimates, which corresponded to large improvements over time and across the intervention (particularly for the PCL-5 and PSYCHLOPS), and minimal increase of symptoms over follow-up. Although inferences should be made cautiously given the small sample size, the 95% CIs for each estimate of d_RM_ from pre to post-intervention excluded zero, and are therefore consistent with statistically significant effects at the *p <*0.05 criterion level. In contrast, the comparable estimates suggested negligible-to-small changes from post-intervention to follow-up, while the 95% CIs all included zero (which was thus a plausible value).

**Figure 3:**
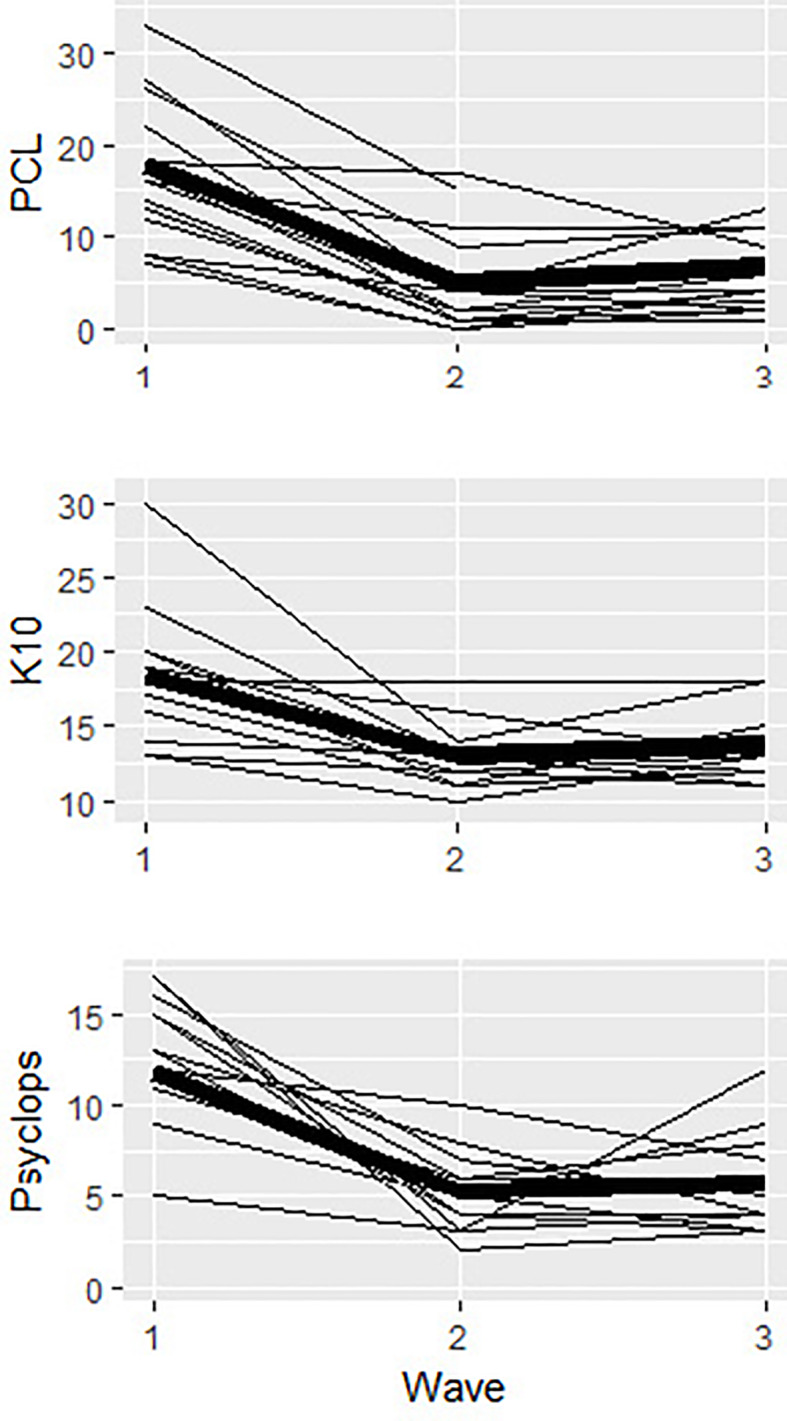
Plots of inidividual trajectories for all outcome measures (K10, PCL-5, Psychlops) across pre-intervention (wave 1), post-intervention (wave 2) and follow-up (wave 3). The main trajectory for each otucome is in bold.

**Figure 3** shows plots of individual trajectories for each outcome measure, with the mean trajectory displayed in bold. As shown, there was discernible variability in change over time, but all trajectories suggested either stable or declining scores from pre to post-intervention. While some trajectories indicated subsequent increases, these slopes were generally modest and reflect follow-up scores that were all still well below pre-intervention levels. As such, there is evidence that the SOLAR program was not associated with a decline in symptom scores.

## Discussion

There is an identified need for a brief intervention that targets individuals who experience difficulties adjusting following disaster and trauma but do not qualify for a formal mental health diagnosis. SOLAR was developed through an international collaboration and informed by existing theoretical and empirical studies regarding mechanisms central to trauma recovery and evidence of effective posttraumatic therapeutic approaches. This study provides preliminary evidence that the SOLAR program is an accessible, brief, and scalable psychosocial intervention that can be delivered by trained frontline workers, including volunteers, professional, and paraprofessional health or disaster workers. While further, more rigorous studies are required, this study suggests that SOLAR has the potential to be useful for individuals with adjustment difficulties following trauma, and holds promise as a complement to existing universal and standard treatment interventions in a strategic, stepped model of mental healthcare. It offers a unique emotional processing component to assist with emotional reactions associated with a traumatic event such as disaster.

Our findings provide preliminary evidence that the SOLAR program can be safely delivered by trained, non-mental health specialists after two days of training delivered by appropriately experienced mental health clinicians. After training, coaches demonstrated improvements in knowledge and confidence in delivering the intervention, and were able to implement the intervention in a safe manner that was acceptable to participants, providing support for the intervention's feasibility. Our findings demonstrate that SOLAR was implementable within existing, diverse, disaster support services in community health and disaster management sectors.

The pilot also provided preliminary evidence that SOLAR is acceptable to disaster survivors in the Australian context, with all participants who were eligible to participate completing all five sessions of SOLAR, and all who responded to open-ended questions concerning program satisfaction stating that the program was useful and that they would recommend it to other disaster survivors. Our findings demonstrated the safety of SOLAR in this particular setting, with no findings of adverse events, and all symptom trajectories trending in a positive direction. In support of the program's efficacy, pre to post-intervention changes demonstrated significant decreases in posttraumatic stress and distress following SOLAR, as well as significant improvements in functioning, with maintenance of improvements over time. In combination, the results warrant further piloting of SOLAR in other post-disaster settings and the conduct of randomized control trials to determine program efficacy.

### Limitations

The findings of the study should be considered alongside the study limitations, which include the small sample size. As such the generalizability of these findings are unknown. We could not look at how gender or culture influenced treatment response, nor did we ask coaches how easy/difficult they found delivering the intervention (which would have informed feasibility). Additionally, the follow-up assessments were conducted three months following the program, and therefore it cannot be determined whether changes that were reported by participants were sustained beyond this time point. Finally, we used traditional formulae for calculation of the dRM, which may overestimate the magnitude of effects given instances of unequal variances.

### Conclusion

SOLAR represents a brief, disaster-focused psychosocial intervention that includes a multi-faceted set of intervention components. It offers an important addition to a disaster mental healthcare response. The current findings suggest that SOLAR is feasible, acceptable, and can be safely administered by non-mental health professionals. Phase III randomized control trials are required to further test its efficacy in other disaster or trauma exposed settings, and further studies are required to test the scalability of the intervention.

## Data Availability Statement

The datasets generated for this study are available on request to the corresponding author.

## Ethics Statement

The studies involving human participants were reviewed and approved by University of Melbourne Health Sciences Human Ethics Sub-Committee, University of Melbourne (HSHEC 1647794). The patients/participants provided their written informed consent to participate in this study.

## Author Contributions

MO'D, AP, DF, RB, JB, SB, WB, AC, MC, DG, NG, BM, AM, CM, JR, PS, JU, PW, SW, and RW contributed to the development of the intervention and designed the evaluation methodology. MO'D, WL, DF, and RB designed the study and interpreted the findings. MO'D, WL, JF, KG, and DF drafted and revised the manuscript. SC conducted the statistical analyses. MO'D, AP, DF, RB, JB, SB, WB, AC, MC, DG, NG, BM, AM, CM, JR, PS, JU, PW, SW, and RW provided expert advice to the manuscript. All authors approved the final version published.

## Funding

This project was funded by Princes Trust Australia, the Australian Commonwealth Government Departments of Defence, Veterans' Affairs, and Health, the University of Melbourne, the Returned Service League (Queensland and Victoria), and the National Health and Medical Research Council Program under Grant Number 1073041.

## Conflict of Interest

The authors declare that the research was conducted in the absence of any commercial or financial relationships that could be construed as a potential conflict of interest.
